# Supplementary material for: Evaluation of a Novel Tool for Screening Inadequate Food Intake in Age-Related Macular Degeneration Patients
Source: Nutrients. 2019 Dec 12;11(12):3031. doi: 10.3390/nu11123031 (PMC6949902; doi:10.3390/nu11123031)
Supplement: Supplementary file 1 [file nutrients-11-03031-s001.pdf]

Scoring criteria for FFQ and SDQ-AMD

Question	Description of measure	Scoring, score = response	Total	Evidence
1a. Vegetables	1 serve = 75g or ½ cup cooked or 1 cup raw salad veg	0= 0-2.99 serves / d<br>1= 3-4.99 serves / d<br>2 ≥ 5 serves /d	2	This question is based on a validated Q in Australia for national monitoring. Measures all vegetables/salads including potato. Increased consumption of vegetables have been associated with protective effects against AMD(1)(2).<br>Cut point is consistent with the Australia Guide to Healthy Eating (AGHE)(3) serve recommendations of 5 serves/d where 1 serve = ½ cup (75g), hence ≥5 serves/d = 375g. Mediterranean diet index tools e.g. MediCul and MEDAS have used similar cut offs; MediCul cut point ≥ 5 serves/d (as per AGHE serves)(4); MEDAS cut point ≥2 serves/d (where 1 serve = 200g, hence ≥400g/d)(5). AGHE recommends 375g/d , which approximates g/d cut point in MEDAS.
1b. Fruit	1 serve = 150g or 1 cup	0 = 0-0.99 serves / d<br>1 = 1-1.99 serves / d<br>2 ≥ 2 serves / d	2	This question is based on a standard question from national surveys in Australia. Cut point includes<br>2 serves/d recommended by the AGHE(3). Cut point allows for higher fruit intake which has been<br>associated with protective effects against AMD(1,2)
1c. Water	1 serve = 250ml	0 = 0-5.99 serves / d<br>1 ≥ 6 serves (1.5L) / d	1	Adequate water intake (1.5-1.9L/d) is important as raised plasma osmolality may be associated with increased risk of age-related retinal diseases(6). Adequate intake is also associated with reduced risk of fatal coronary heart disease and dehydration with impaired cognitive performance (7–9) Australian Nutrient Reference Values for adequate intake of water in adults 50+ years of age are 2.1L/d for women, and 2.6L/d for men(10)
2a. Red meat	1 serve = 100-150g Includes beef, veal, lamb, pork, kangaroo	0 = <0.5 serves/wk; >4.5 serves/wk<br>1 = 3-4.5 serves/wk<br>2 = 0.5-2.99 serves / wk	2	Fresh red meat intake is associated with a dose-response increase in odds of AMD when consumed ≥ 3 times(11). However, this study adjusted for zinc, which is found in high concentrations in the retina, and included in the AREDS formulation to help reduce the risk of AMD progression(12). Serves are based on commonly consumed portions (100-150g) in Australia rather than the 65g as defined in the AGHE(3).

				AGHE recommends ≤ 455g (3-4.5 serves)/week, and the Australian Heart Foundation recommends ≤ 350g (2.5-3.5 serves)/week(13). Other diet tools have included different cut points e.g. MedDietScore (14) uses cut point ≤1/wk; MEDLIFE(15) uses cut point <2/wk; MIND diet (16) allows higher exposure to red meat and meat products <4 meals/wk.
2b. Processed meat	1 serve = 100-150g e.g. 2 thinner sausages, 4 slices processed meat; 1 meat pie	0 = >1 serves / wk<br>1 = 0.5-1 serves / wk<br>2 = 0-<0.5 serves / wk	2	Processed meat (as with high red meat intake) has been associated with a dose-response increased risk of early AMD when >1 serve consumed, with salami/ continental sausage in particular showing strong association with early and late AMD(11).<br>Processed meat is also strongly associated with chronic disease risk and mortality(6).<br>Cut point is consistent with MEDLIFE ≤ 1 serve/wk (15)and 0 times/wk by *Ciccarone E et al. (2003)*(17). Goulet *et al.* used cut point <1 portion (50-100g)/wk(18).<br>This question uses a similar serve size to that for fresh red meat.
2d. Fish	1 serve = 1 small fillet or tin of fish or 200g seafood	0 = 0-0.99 serves / wk<br>2 = 1-1.99 serves / wk<br>4 = ≥2 serves / wk	4	Fish intake has been associated with a reduced risk of AMD, benefits seen with at least 1 serve/wk with greater benefits found with increasing regular consumption(19). Higher intakes of omega 3 fatty acids have shown an inverse relationship with AMD onset (20), with evidence that higher proportion of DHA to EPA being protective against AMD(21).<br>The Heart Foundation recommends 2-3 serves oily fish/wk for all Australians(13)
3. Legumes	1 serve = 1 cup cooked or 150g	0 = 0-0.99 serves / wk<br>1 = 1-1.99 serves / wk<br>2 = ≥2 serves / wk	2	Lower GI diets (including legumes) have been associated with a reduced risk of AMD onset and progression(21–23)<br><br>GLNC recommends legumes are consumed 2-3 times/wk(24). AGHE protein serve (1 cup cooked) rather than vegetable serve (1/2 cup cooked) has been used for legumes as it is more consistent with Australian portions(3)
4. Nuts	1 serve = 30g or 1.5 tablespoons of nut spread	0 = <1 serves / wk<br>1 = 1-2.49 serves / wk<br>2 ≥ 2.5 serves / wk	2	Nut consumption as part of a Meditteranean diet has been shown to be protective against AMD(1), with 1 or more servings per week compared with no intake has also been associated with a decreased risk of AMD progression and may be related to the fatty acid content of nuts,

				although evidence about nut consumption and risk of AMD is limited(19).<br>Mediterranean diet index tools e.g. MEDAS(5) and MediCul(4) have allowed for cut-points of ≥ 3 serves/week due to observational studies showing greater CVD protections with exposure to nuts ≥5 serves/wk. Goutlet et al uses a cut point >2 portions/d for both legumes and nuts/seeds(18).<br>AGHE(3) recommends 2 serves/d for women and 2.5 serves/d for men of meat/meat alternatives e.g. nuts (1 serve nuts = 30g).
5. Green vegetables		Only score the dark green leafy type (second part of q.)<br>0 = 0-1.99 times / wk<br>1 = 2-3.99 times / wk<br>2 = ≥4 times/ wk	2	Dark green leafy vegetables are rich in carotenoids (e.g. lutein (which are asssociated with decreased risk of wet AMD. Recommended intake of at least 2-4 serves/wk for carotenoid benefits with increased benefits when consuming up to 2 serves/d (19). In addition to lutein, dark green leafy vegetables also contain nitrate and vitamin K, which provide vascular and cognitive benefits(25,26). Two questions are asked about different types of green vegetables to minimise response bias. Cut point relates to frequency of exposure for most days per week, which is simpler to estimate than quantity consumed. This question and cut-points are similarly used in MediCul(4).
6. Eggs		0 = <1 times / wk; > 7 times/ wk<br>1 = >1-1.99 times / wk; >4-7 times/ wk<br>2 = 2-4 times / wk	2	Eggs are a bioavailable source of dietary carotenoids (lutein/zeaxanthin)(27). Moderate egg consumption (2-4 eggs/wk) shown to signficantly reduce risk of progression to late AMD after 15 years. Some evidence of protection when 5-6 eggs/wk consumed after 10 years(28).<br>Heart Foundation Australia recommends <7 eggs/wk for people with high cholesterol, heart disease of type 2 diabetes(29).<br>Allowance for up to and including 7 eggs for 1 score for those who do not have the above health problems.
7a. Wholegrains or lower GI (bread as proxy)		0 = white > wholegrain + wholemeal + rye + sourdough;	2	Lower GI diets (e.g. grain bread, sourdough) have been associated with a reduced risk of AMD onset and progression(21–23).

		1 = white is equal to wholegrain + wholemeal + rye + sourdough;<br>2 = white < wholegrain + wholemeal + rye + sourdough		This question uses bread as a proxy for quality of grains most frequently consumed. Relative frequency is used as it is easier to rate than quantities.
7b. Lower GI cereals		0 = higher GI ≥ lower GI<br>1 = lower GI > higher GI	1	Lower GI diets (e.g. grain bread, sourdough) have been associated with a reduced risk of AMD onset and progression(21–23).<br>This question uses bread as a proxy for quality of grains most frequently consumed. Relative frequency is used as it is easier to rate than quantities.
8a. Biscuits and cakes, ice cream, sugary drinks, takeaway, processed potato		0 = >1 times /d<br>1 = >0.5 – 1 times /d<br>2 = ≤0.5 times /d	2	AGHE allows for discretionary choices in taller or more active people of maximum 2.5 serves/day in men aged 50+ and women up to 70 years of age, and maximum 2 serves/d in women aged 70+ (3). As processed meats and alcohol are not included in this section (and other discretionary items not included in the questionnaire) the cut-offs for these items have been lowered based on the AHS 2011-12(30) where total discretionary foods made up 35% of total energy, with biscuits and cakes (24.3%), ice cream (4.3%), sugary drinks (5.4%), processed potato (as a proxy for takeaway food as well) (4.9%). As these percentages add up to roughly 40% of total discretionary foods, 40% of 2 serves/day = 0.8 serves/day. As AHS' 35% of energy from discretionary foods is high, 40% was applied to the lower AGHE max. recommendation and rounded down for the 0.5 serves/day cut-off.
9. Alcoholic beverages	*Note: convert drinks to "standard drinks" before applying rules, see below**	0= >14 standard drinks per week (from question 9b)<br><br>1= >7-14 standard drinks per week of	2	Cut point of 7 standard drinks/wk (i.e.1 standard/d) as alcohol consumption of 2 or more standard drinks/d is linked with increased risk of AMD regardless of alcohol type or gender(31–33).<br>Australian guidelines recommend no more than 2 standard drinks/d, where 1 standard drink is 10g alcohol(34)

		any alcohol (from question 9b)<br><br>2= NON-DRINKERS (from question 9a) OR<br>2= 0 to 7 standard drinks per week		
10. Fat types		0 = Others total > olive oil<br>1 = Others total = olive oil<br>2= Others total < olive oil	2	ALIENOR study has shown protective effects of olive oil consumption for late AMD and did not find any associations with other fat sources(35). Olive oil is also linked to improving the bioavailability of lutein in lutein-deficient mice(36).<br><br>Relative frequency of exposure to various fat types used as it is easier to rate than quantifying amounts. This is similarly used in the MediCul tool(4)
**TOTAL**			**30**	

***Standard Drink Conversions**

Full Strength Beer (375 ml) =	*1.4*	*standard drinks*
Lite Beer (375 ml) =	*0.8*	*standard drinks*
Red Wine (150 ml) =	*1.6*	*standard drinks*
White Wine/champagne (150 ml) = 1.4	*standard drinks*	
Spirits (30 ml) =	*1*	*standard drinks*

Possible screening description:
21-30 / 30 = Excellent
16- 20 / 30 = Good. Keep going!
10- 15 / 30 = More room to improve
<10 / 30 = It's time to take action!

References

1. Nunes S, Alves D, Barreto P, Raimundo M, da Luz Cachulo M, Farinha C, et al. Adherence to a Mediterranean diet and its association with age-

related macular degeneration. The Coimbra Eye Study-Report 4. Nutrition. 2018/03/17. 2018;51–52:6–12.

2. Kim EK, Kim H, Kwon O, Chang N. Associations between fruits, vegetables, vitamin A, beta-carotene and flavonol dietary intake, and age-related macular degeneration in elderly women in Korea: the Fifth Korea National Health and Nutrition Examination Survey. Eur J Clin Nutr. 2017/09/28. 2018;72(1):161–7.
3. National Health and Medical Research Council. Dietary Guidelines for Australian Adults. Canberra, Commonwealth of Australia; 2003.
4. Radd-Vagenas S, Fiatarone Singh MA, Daniel K, Noble Y, Jain N, O'Leary F, et al. Validity of the Mediterranean Diet and Culinary Index (MediCul) for Online Assessment of Adherence to the "Traditional" Diet and Aspects of Cuisine in Older Adults. Nutrients. 2018/12/07. 2018;10(12).
5. Schröder H, Fitó M, Estruch R, Martínez-González MA, Corella D, Salas-Salvadó J, et al. A short screener is valid for assessing Mediterranean diet adherence among older Spanish men and women. J Nutr [Internet]. 2011 Jun [cited 2019 Oct 29];141(6):1140–5. Available from: http://www.ncbi.nlm.nih.gov/pubmed/21508208
6. Bringmann A, Hollborn M, Kohen L, Wiedemann P. Intake of dietary salt and drinking water: Implications for the development of age-related macular degeneration. Mol Vis [Internet]. 2016;22:1437–54. Available from: https://www.ncbi.nlm.nih.gov/pubmed/28031693
7. Chan J, Knutsen SF, Blix GG, Lee JW, Fraser GE. Water, other fluids, and fatal coronary heart disease: The Adventist health study. Am J Epidemiol. 2002 May 1;155(9):827–33.
8. Lieberman HR. Hydration and cognition: a critical review and recommendations for future research. J Am Coll Nutr [Internet]. 2007 Oct [cited 2019 Oct 29];26(5 Suppl):555S-561S. Available from: http://www.ncbi.nlm.nih.gov/pubmed/17921465
9. Wilson M-MG, Morley JE. Impaired cognitive function and mental performance in mild dehydration. Eur J Clin Nutr [Internet]. 2003 Dec [cited 2019 Oct 29];57 Suppl 2:S24-9. Available from: http://www.ncbi.nlm.nih.gov/pubmed/14681710
10. Ministry of Health; Nutrient Reference Values: Water [Internet]. 2014 [cited 2019 Oct 29]. Available from: https://www.nrv.gov.au/nutrients/water
11. Chong EW-T, Simpson JA, Robman LD, Hodge AM, Aung KZ, English DR, et al. Red Meat and Chicken Consumption and Its Association With Age-related Macular Degeneration. Am J Epidemiol [Internet]. 2009;169(7):867–76. Available from: https://doi.org/10.1093/aje/kwn393
12. Age-Related Eye Disease Study Research Group. A randomized, placebo-controlled, clinical trial of high-dose supplementation with vitamins C and E, beta carotene, and zinc for age-related macular degeneration and vision loss: AREDS report no. 8. Arch Ophthalmol. 2001/10/23. 2001;119(10):1417–36.
13. Heart Foundation; Meat, poultry and seafood [Internet]. 2019 [cited 2019 Oct 29]. Available from: heartfoundation.org.au/healthy-eating/food-and-nutrition/protein-foods/meat-poultry-and-seafood
14. Panagiotakos D, Kalogeropoulos N, Pitsavos C, Roussinou G, Palliou K, Chrysohoou C, et al. Validation of the MedDietScore via the determination of plasma fatty acids. Int J Food Sci Nutr [Internet]. 2009 [cited 2019 Oct 29];60 Suppl 5:168–80. Available from: http://www.ncbi.nlm.nih.gov/pubmed/19353422
15. Sotos-Prieto M, Moreno-Franco B, Ordovás JM, León M, Casasnovas JA, Peñalvo JL. Design and development of an instrument to measure overall lifestyle habits for epidemiological research: the Mediterranean Lifestyle (MEDLIFE) index. Public Health Nutr [Internet]. 2015 Apr [cited 2019 Oct 29];18(6):959–67. Available from: http://www.ncbi.nlm.nih.gov/pubmed/25025396

16. Morris MC, Tangney CC, Wang Y, Sacks FM, Barnes LL, Bennett DA, et al. MIND diet slows cognitive decline with aging. Alzheimer's Dement. 2015 Sep 1;11(9):1015–22.
17. Ciccarone E, Di Castelnuovo A, Salcuni M, Siani A, Giacco A, Donati MB, et al. A high-score Mediterranean dietary pattern is associated with a reduced risk of peripheral arterial disease in Italian patients with Type 2 diabetes. J Thromb Haemost [Internet]. 2003 Aug [cited 2019 Oct 29];1(8):1744–52. Available from: http://www.ncbi.nlm.nih.gov/pubmed/12911588
18. Goulet J, Lamarche B, Nadeau G, Lemieux S. Effect of a nutritional intervention promoting the Mediterranean food pattern on plasma lipids, lipoproteins and body weight in healthy French-Canadian women. Atherosclerosis. 2003 Sep 1;170(1):115–24.
19. Broadhead GK, Grigg JR, Chang AA, McCluskey P. Dietary modification and supplementation for the treatment of age-related macular degeneration. Nutr Rev. 2015/06/18. 2015;73(7):448–62.
20. SanGiovanni JP, Chew EY, Clemons TE, Davis MD, Ferris 3rd FL, Gensler GR, et al. The relationship of dietary lipid intake and age-related macular degeneration in a case-control study: AREDS Report No. 20. Arch Ophthalmol. 2007/05/16. 2007;125(5):671–9.
21. Chiu CJ, Klein R, Milton RC, Gensler G, Taylor A. Does eating particular diets alter the risk of age-related macular degeneration in users of the Age-Related Eye Disease Study supplements? Br J Ophthalmol. 2009/06/11. 2009;93(9):1241–6.
22. Kaushik S, Wang JJ, Flood V, Tan JS, Barclay AW, Wong TY, et al. Dietary glycemic index and the risk of age-related macular degeneration. Am J Clin Nutr. 2008/10/10. 2008;88(4):1104–10.
23. Chiu C-J, Milton RC, Klein R, Gensler G, Taylor A. Dietary carbohydrate and the progression of age-related macular degeneration: a prospective study from the Age-Related Eye Disease Study. Am J Clin Nutr [Internet]. 2007;86(4):1210–8. Available from: https://doi.org/10.1093/ajcn/86.4.1210
24. Grains and Legumes Nutrition Council. Lifting the lid on legumes: the benefits of choosing legumes [Internet]. 2013 [cited 2019 Oct 29]. Available from: https://www.glnc.org.au/wp-content/uploads/2011/04/GLN_LiftingtheLid_WEB.pdf
25. Mohn ES, Johnson EJ. Lutein and Cognition Across the Lifespan. Nutr Today. 2017 Jul 1;52(4):183–9.
26. Lidder S, Webb AJ. Vascular effects of dietary nitrate (as found in green leafy vegetables and beetroot) via the nitrate-nitrite-nitric oxide pathway. Br J Clin Pharmacol. 2013 Mar;75(3):677–96.
27. Eisenhauer B, Natoli S, Liew G, Flood VM. Lutein and Zeaxanthin-Food Sources, Bioavailability and Dietary Variety in Age-Related Macular Degeneration Protection. Nutrients. 2017/02/18. 2017;9(2).
28. Gopinath B, Liew G, Tang D, Burlutsky G, Flood VM, Mitchell P. Consumption of eggs and the 15-year incidence of age-related macular degeneration. Clin Nutr. 2019/03/28. 2019;
29. Heart Foundation; Eggs [Internet]. [cited 2019 Oct 29]. Available from: https://www.heartfoundation.org.au/healthy-eating/food-and-nutrition/protein-foods/eggs
30. Australian Bureau of Statistics. Australian Health Survey: Nutrition First Results - Foods and Nutrients, 2011-12: Discretionary Foods [Internet]. 2014. Available from: https://www.abs.gov.au/ausstats/abs@.nsf/Lookup/by Subject/4364.0.55.007~2011-12~Main Features~Discretionary foods~700
31. Adams MK, Chong EW, Williamson E, Aung KZ, Makeyeva GA, Giles GG, et al. 20/20--Alcohol and age-related macular degeneration: the Melbourne Collaborative Cohort Study. Am J Epidemiol. 2012/08/01. 2012;176(4):289–98.

32. Chong EW, Kreis AJ, Wong TY, Simpson JA, Guymer RH. Alcohol consumption and the risk of age-related macular degeneration: a systematic review and meta-analysis. Am J Ophthalmol. 2008/02/05. 2008;145(4):707–15.
33. Fraser-Bell S, Wu J, Klein R, Azen SP, Varma R. Smoking, alcohol intake, estrogen use, and age-related macular degeneration in Latinos: the Los Angeles Latino Eye Study. Am J Ophthalmol. 2006/01/03. 2006;141(1):79–87.
34. Australian Government Department of Health. How much alcohol is safe to drink? [Internet]. 2019 [cited 2019 Oct 29]. Available from: https://www.health.gov.au/health-topics/alcohol/about-alcohol/how-much-alcohol-is-safe-to-drink
35. Cougnard-Grégoire A, Merle BMJ, Korobelnik J-F, Rougier M-B, Delyfer M-N, Le Goff M, et al. Olive Oil Consumption and Age-Related Macular Degeneration: The Alienor Study. PLoS One [Internet]. 2016;11(7):e0160240. Available from: https://doi.org/10.1371/journal.pone.0160240
36. Nidhi B, Mamatha BS, Baskaran V. Olive oil improves the intestinal absorption and bioavailability of lutein in lutein-deficient mice. Eur J Nutr [Internet]. 2014 Feb [cited 2019 Oct 29];53(1):117–26. Available from: http://www.ncbi.nlm.nih.gov/pubmed/23543147
